# A DIY Fabrication Approach for Ultra-Thin Focus-Tunable Liquid Lens Using Electrohydrodynamic Pump

**DOI:** 10.3390/mi12121452

**Published:** 2021-11-26

**Authors:** Taichi Murakami, Yu Kuwajima, Ardi Wiranata, Ayato Minaminosono, Hiroki Shigemune, Zebing Mao, Shingo Maeda

**Affiliations:** 1 Department of Mechanical Engineering, Shibaura Institute of Technology, Tokyo 135-8548, Japan; nd21105@shibaura-it.ac.jp (Y.K.); nb19501@shibaura-it.ac.jp (A.W.); nb20108@shibaura-it.ac.jp (A.M.); 2 Department of Mechanical and Industrial Engineering, Faculty of Engineering, University of Gadjah Mada, Jalan Grafika No. 2, Yogyakarta 55281, Indonesia; 3 Department of Electrical Engineering, Shibaura Institute of Technology, Tokyo 135-8548, Japan; hshige@shibaura-it.ac.jp

**Keywords:** electro hydro dynamics, lens, soft actuator, EHD pump

## Abstract

Demand for variable focus lens is increasing these days due to the rapid development of smart mobile devices and drones. However, conventional mechanical systems for lenses are generally complex, cumbersome, and rigid (e.g., for motors and gears). This research proposes a simple and compact liquid lens controlled by an electro hydro dynamics (EHD) pump. In our study, we propose a do-it-yourself (DIY) method to fabricate the low-cost EHD lens. The EHD lens consists of a polypropylene (PP) sheet for the exterior, a copper sheet for the electrodes, and an acrylic elastomer for the fluidic channel where dielectric fluid and pure water are filled. We controlled the lens magnification by changing the curvature of the liquid interface between the dielectric fluid and pure water. We evaluated the magnification performance of the lens. Moreover, we also established a numerical model to characterize the lens performance. We expect to contribute to the miniaturization of focus-tunable lenses.

## 1. Introduction

Recently, the development of smart mobile devices, automobiles, and drones has dramatically increased the demand for small cameras [1,2]. Conventional miniature cameras consist of a complementary metal-oxide semiconductor (CMOS) image sensor, an infrared (IR) filter, a micro-actuator, and an optical lens. For conventional lenses, changing the position of the solid glass lens relies on the change of the focal length [3]. Conventional electromechanical lenses have generally complex structures since they require additional dynamic parts (e.g., motors and gears to move the lenses, retractable solid lenses). To overcome these shortcomings, researchers have proposed various types of liquid-variable lenses [4,5,6,7,8,9,10,11,12]. The focal length of liquid-variable lenses, which affects the magnification of the lenses, can be adjusted by changing the refractive index and surface shape of the medium. To actuate the liquid lenses, researchers have proposed various approaches, e.g., elasticity of the materials [4,5,6], electrowetting effect [7,8], dielectrophoretic effect [9,10], magnetic actuation [11,12], and stimuli-responsive hydrogels [13,14]. Lenses made by elastic membranes feature as variable focus due to their high hermeticity. However, it is not easy to replace the liquid in the lens part, and there is no versatility in performance. Elastic membranes, such as polydimethylsiloxane (PDMS), shrink and swell when they come into contact with the liquids. These factors complicate the refractive index of the lenses. Many lenses using electrowetting or dielectric effect contain two immiscible liquids of oil and water to form an interface, whose shapes are decided by the applied voltages. Lenses that use electrowetting deform the shape of the interface by the static electrical force. In the dielectric lenses, the generated dielectric force controls the shape of the interface. These lenses are open to the liquid, making it easy to replace the liquid, limiting the placing angles of the device. Liquid lenses using magnetic actuation can achieve a large focus. However, the equipment is heavy and requires expensive accessories to drive the magnets and electromagnets. 

To couple with the aforementioned problems, we aim to develop a simple and low-cost, compact lens driven by an electro hydro dynamics (EHD) pump (EHD lens). Our device is also focus tunable. We utilize a DIY approach for the EHD lenses by adopting simple and reliable digital fabrication (laser cutter, cutting plotter). A DIY approach is helpful in developing focus-tunable lenses since the demand of the small cameras increases. The DIY approach has been previously reported as a reliable method for the fabrications of soft actuators such as dielectric elastomer actuators (DEAs) [15,16,17] and EHD pumps [18,19]. The DIY method is a process in which a non-professionally certified person builds or repairs something by themselves [20]. The DIY process allows a researcher to freely customize the equipment based on their design requirements [21]. By implementing the DIY methods in the fabrication of focus-tunable lenses, we expect that the approach will contribute to the fast development and innovation of compact focus-tunable lenses. Moreover, our EHD lens is capable of tuning the focus by changing the curvature of the liquid interface driven by the EHD pump. Our goal is to design the device with the following criteria: (1) small, thin, and lightweight; (2) composed of inexpensive components and easily fabricated without specialized knowledge; (3) replaceable in liquid of the lens part for performance enhancement; and (4) drivable at the various tilt angles. Finally, we show our system’s integration with a smartphone as a demonstration for an everyday scenario.

## 2. EHD Pump to Focus-Tunable Liquid Lens

### 2.1. EHD Pump

**Figure 1:**
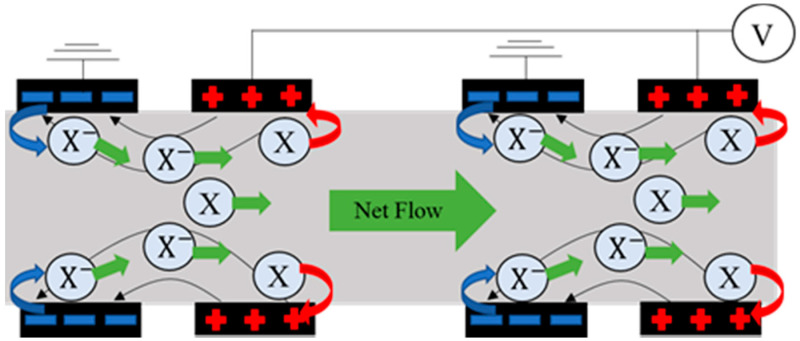
Driving mechanism of EHD pump.

EHD works due to the interaction between an electric field and a flow field through an insulating fluid [22,23,24]. Figure 1 describes the principle of EHD pumping. The mechanism is charge injection, which is a field-emission-based phenomenon. Figure 1 shows the electrode arrangement employed in this device. If the electric field is sufficiently high, it can move directly from the cathode surface into the dielectric liquid. The ions (X-) thus formed are accelerated by electrophoretic forces and impart momentum to the neutral liquid molecules until the ions (X-) are discharged at the anode. These mechanisms can create a net flow with a planar electrode arrangement and a DC electric field. The direction of the flow is reversible by switching the polarity of the electric field.

### 2.2. EHD Lens

**Figure 2:**
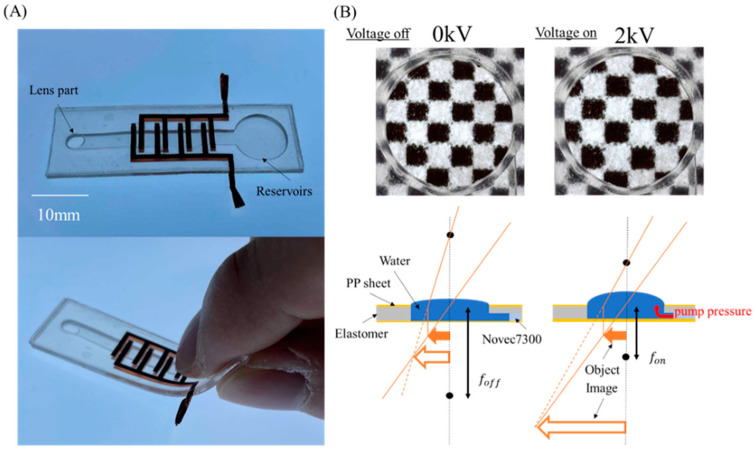
(**A**) Focus-tunable lens powered by an EHD pump. (**B**) Schematic diagram of focus-tunable lens driven by EHD pump.

Figure 2A shows our focus-tunable EHD lens and the design of the EHD lens is very simple: the EHD pump is assembled with a lens part and a liquid reservoir sealed by a thin layer of diaphragm. Moreover, we adjusted the diameter of the lens part and flow path to facilitate various driving positions even if the lens part is open using surface tension. We used a simple and reliable digital fabrication (laser cutter and cutting plotter) method to make the EHD lens. In addition, the copper tape was used for the electrodes [18]. 

The schematic of the EHD lens is shown in Figure 2B. We filled the flow path in the EHD lens with insulating fluid (3M, Novec HFE7300), and the enlarged part with pure water. We defined the focal length of the lens under the power-off state as $f_{off}$ and the focal length after power-on as $f_{on}$. The lens part has a small curvature in the initial state, so an imaginary image is visible. When we applied the voltage, the check pattern was enlarged as shown in Figure 2B. This is because driving the EHD pump raises the liquid level, therefore changing the radius of curvature of the lens part. As a result, the focal length decreased, which realized a larger imaginary image.

**Figure 3:**
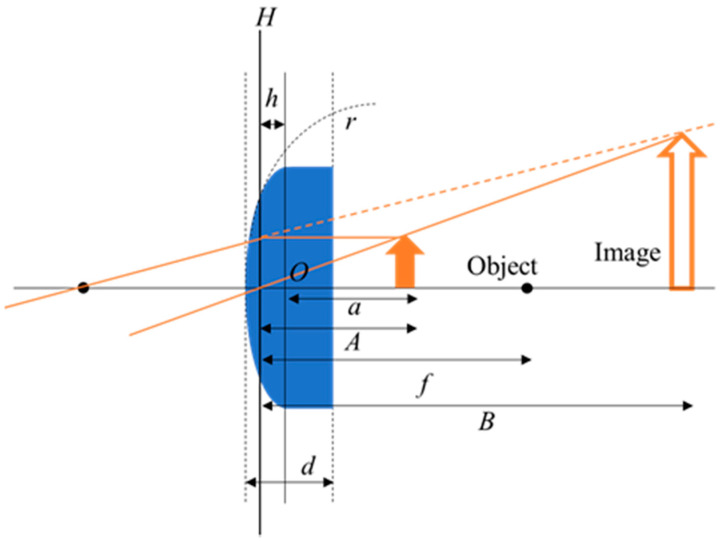
Model diagram of the plano-convex lens.

Similar to a thick plano-convex lens, the EHD lens is shown in Figure 3. A plano-convex lens has a radius of infinite curvature on one side. Therefore, the relationship between the radius of curvature *r* [mm] and the focal length *f* [mm] is shown as in Equation (1) [11,25]. The origin is the intersection point of the lens’s optical axis and the plane of the device. The distance *h* [mm] from the principal plane *H* to the origin is shown in Equation (2). When we place the object directly behind the lens, the distance between the origin and the object is *a* = 1.3 mm; since the PP sheet is very thin (0.15 mm thick), Equation (4) is valid for our case when the refractive index is air [23]. The focal length *f* [mm] is the distance between the principal plane and the focal point, *A* [mm] is the distance between the principal plane and the object, and *B* [mm] is the distance between the principal plane and the image. *N* is the refractive index of the liquid in the lens. The refractive index of pure water is 1.33 [26]. In this case, the magnification *m* of the lens is shown as Equation (6) [27]. Since the magnification *m* of a lens is expressed in terms of line ratio, the area magnification is $m^{2}$ when the object is a circle.

(1) $$f=\frac{r}{N-1},\;$$

(2) $$h=\frac{fd\left(N-1\right)}{Nr},$$

(3) A=a+h,

(4) $$\frac{1}{A}+\frac{1}{B}=\frac{1}{f},$$

(5) $$m=\left|\frac{B}{A}\right|.$$

## 3. Materials and Methods

**Figure 4:**
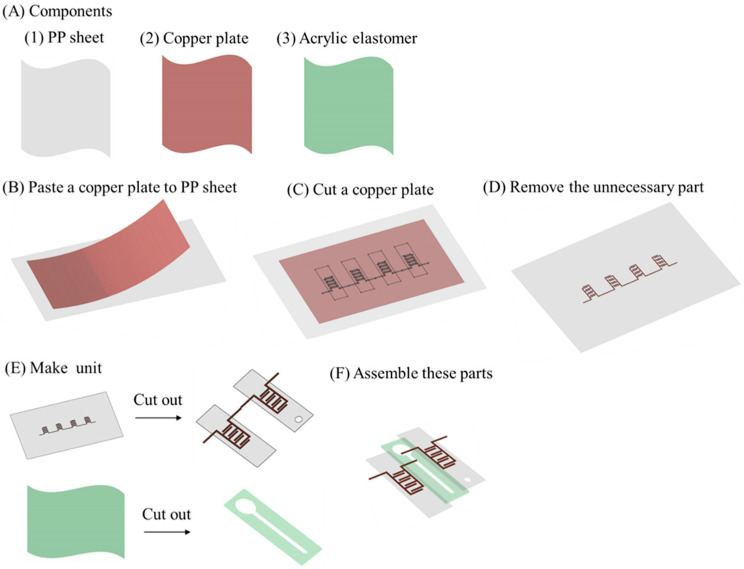
Fabrication methods for focus-tunable lens driven by EHD pump. (**A**) Main components used in the EHD lens. (**B**) A copper plate is pasted onto the PP sheet. (**C**) Cutting into the copper plate. (**D**) Removing the unnecessary part. (**E**) Making units. (**F**) Assemble to complete the EHD lens.

Figure 4 shows the fabrication procedure of the EHD lens. Figure 4A shows the three main components used in the EHD lens. The exterior was made of polypropylene (PP) sheet, and the electrodes were made of copper plate with a thin layer adhesive underneath. Copper, well-known for its high electrical conductivity, is often used for the electrodes of dielectric actuators. An acrylic elastomer (3M VHB4910J) with excellent electrical insulation and adhesive properties was used for the intermediate layer. First, we pasted a copper plate onto the PP sheet (Figure 4B). Next, we used a cutting plotter (GRAPHTEC CE6000-40 Plus) to cut into the copper sheet (Figure 4C). After removing the unnecessary parts, we used a laser cutter (TROTEC Speedy 100) to separate the copper sheet into individual units (Figure 4D,E). Next, we also manufactured the channel by the laser cutter (TROTEC Speedy 100) (Figure 4E). Finally, we assembled all the parts to complete the EHD lens (Figure 4F).

To drive the EHD lens with a low voltage, the height of the flow channel needs to be reduced. However, when the height is reduced, the fluid flow becomes difficult due to fluid effects such as viscous resistance. Therefore, we set the height to 1 mm, referring to the existing EHD pump [24]. This fabrication method is time-saving and cost-effective and it can be fabricated at about 8 EHD lenses per hour at a cost of less than $7 per lens. The thickness and weight of the EHD lens are 1.3 mm and 0.85 g, respectively.

**Figure 5:**
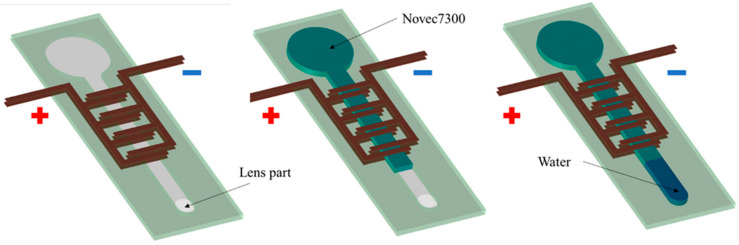
Experimental setup of focus-tunable lens driven by EHD pump.

Figure 5 shows the liquid filling process of the EHD lens. First, we injected 0.15 mL of insulating fluid (3M, Novec HFE7300) into the flow channel of the made EHD lens using a syringe. Next, we injected 0.03 mL of pure water into the lens part using a syringe. Note that air bubbles ought to avoid entering the flow channel of the EHD lens. After injecting the insulating fluid into the EHD lens, we gently rubbed the EHD lens to release the bubbles. Then, we set the object directly behind the lens part and applied a DC voltage to magnify the object. At this time, the EHD lens had to be driven in the direction that pushes up the droplets in the lens part, so we paid attention to the direction of the electrode. 

## 4. Results and Discussion

### 4.1. Image Focus Test of EHD Lens

**Figure 6:**
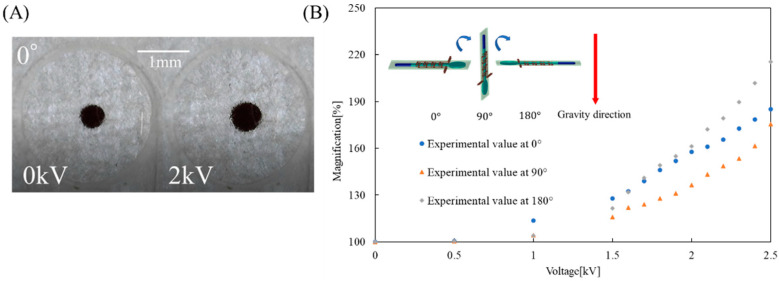
Image focus test. (**A**) Image focus test when the EHD lens is at 0°. (**B**) Results of image focus test at 0°, 90°, and 180°.

To evaluate the performance of the EHD lens, we applied a DC voltage to the EHD lens and the magnification of the object was measured (Figure S1 shows the complete experimental setup for the device performance investigation). We placed the object directly behind the lens part. In order to investigate the effect of gravity on the performance of the EHD lens, we conducted experiments when the EHD lens was placed at 0°, 90°, and 180° to the ground, respectively. In detail, when the EHD lens was at 0°, the EHD lens magnified the object as shown in Figure 6A. We used ImageJ (ImageJ is software that has an image processing function [28]) to analyze the image and calculate the area of the object. Figure 6B shows the area magnification of the object versus the applied voltage. The vertical axis shows the area magnification of the object and the horizontal axis shows the applied voltages. Here, the area magnification indicates the percentage of the area of the image at 0 kV.

As the voltage increases, the radius of curvature of the lens changes and the area magnification of the object increases. At 0°, the EHD lens enlarged the object by about 185% at 2.5 kV applied. At 90°, the EHD lens enlarged the object by about 175% at 2.5 kV applied. The maximum magnification at 90° was about 10% smaller than that at 0°. At 180°, the EHD lens enlarged the object by about 215% at 2.5 kV. At 2.5 kV, the maximum magnification at 180° was about 30% larger than that at 0°. At 2.5 kV, the maximum magnification at 180° was about 30% larger than that at 0°. This result suggests that not only pump pressure but also gravity contributed to the deformation of the lens. When we applied a voltage of 2.5 kV or higher, the drive became unstable due to the overflow of droplets in the lens, or the electrical breakdown of the EHD lens.

**Figure 7:**
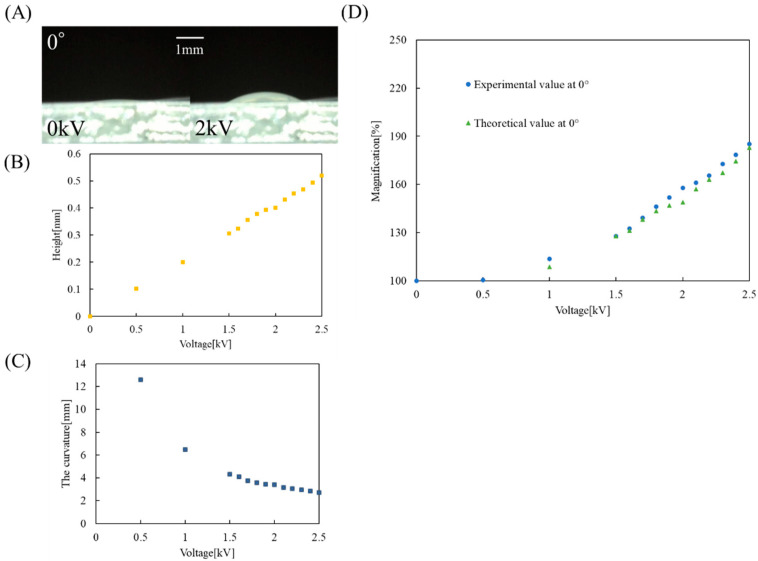
(**A**) Droplet height when the EHD lens is at 0°. (**B**) Results of droplet height in image focus test. (**C**) Results of the curvature of the lens when changing the voltage. (**D**) Comparison of experimental and theoretical values for focus test at 0°.

Next, we calculated the theoretical values for the 0° case. Figure 7A shows the interface height of the lens part at 0° when we applied the voltage at 0 kV and 2 kV, respectively. Figure 7B indicates the liquid level height of the lens part versus applied voltage for the 0° case. From the results of Figure 6B, the area $M_{1}$ of the image at 0 kV is 0.256${\;\mathrm{mm}}^{2}$, and the area $M_{o}$ of the object is 0.211${\;\mathrm{mm}}^{2}$. Therefore, the area magnification $m_{1}$ of the image at 0 kV for the object is 1.21. From the result of Figure 7B, we calculated the area magnification $m_{2}$ of the image after applying voltage to the object. Using the Newton–Raphson method [29], we fixed the lens diameter R at 3.2 mm, and calculated the radius of curvature *r* [mm] from the height of the liquid surface. Figure 7C indicates the results of the curvature of the lens when changing the voltage. Next, we derived the focal length *f* [mm] using Equation (1). At this time, we placed the object directly behind the lens, so *a* = 1.30 mm. We used Equations (2)–(5) to calculate the area magnification factor $m^{2}$ = $m_{2}$. When the area of the image after applying voltages is $M_{2}\left[{\mathrm{mm}}^{2}\right]$, the area magnification $m_{3}$ of the image after applying a voltage to the area of the image at 0 kV is shown in Equation (6). Figure 7D shows a comparison between the experimental value of the EHD lens at 0° and the theoretical value of $m_{3}$ as described in Equation (6).

(6) $$m_{3}=\frac{M_{2}}{M_{1}}=\frac{m_{2}M_{o}}{m_{1}M_{o}}=\frac{m_{2}}{m_{1}}.$$

Figure 7D shows that the theoretical value is slightly lower than the experimental value due to a slight misalignment between the lens’s optical axis and the center of the object.

### 4.2. Extended Performance of the EHD Lens

In the previous literature, the oil phase liquids with a high refractive index such as dimethyl silicone oil (*n* = 1.60) or glycerine (*n* = 1.47) were preferred to improve the performance of the EHD lens [30,31]. 

**Figure 8:**
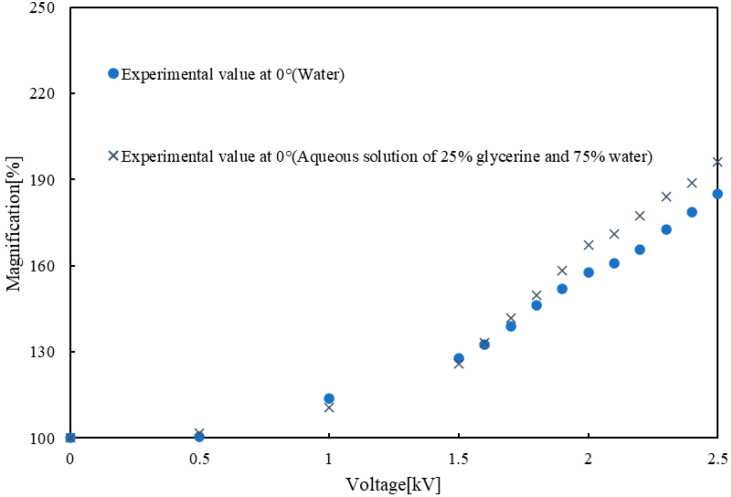
Comparison of experimental results in the case of water and glycerine solution.

However, due to the low density of dimethyl silicone oil and glycerine, their surface tension is low and could not form droplets. Therefore, we used an aqueous solution of 25% glycerine and 75% water (*n* = 1.40) to investigate the area magnification when the tilt angle is set to 0° [30]. Figure 8 shows a comparison of the experimental results of two groups: one group using water and one with glycerine solution as the oil phase fluid. The results show that the maximum magnification in the group with glycerine solution was about 11% larger than that of the group with pure water when the applied voltage is 2.5 kV. The EHD lens was able to achieve the larger area magnification by using a solution with a higher refractive index. However, since the surface tension of glycerine solution was lower than that of pure water, the interface between the oil phase and water phase became unstable at 90° and 180°. Thus, it is easy to extend the performance of the EHD lens by changing the liquid in the lens part depending on the intended use.

### 4.3. Demonstration of the EHD Lens

**Figure 9:**
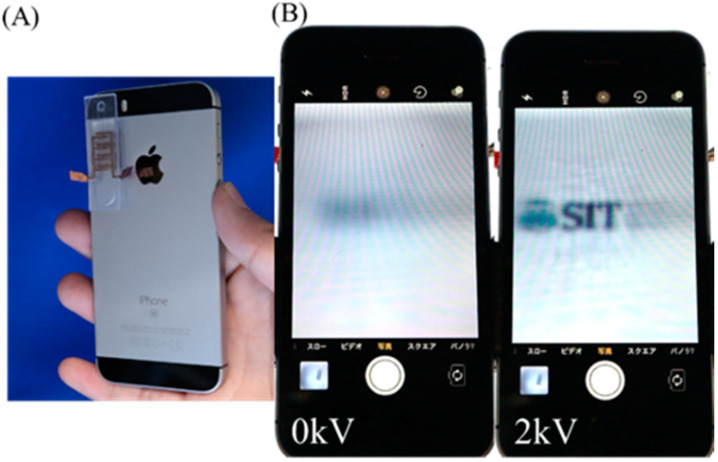
EHD lens demonstration with a smartphone. (**A**) EHD lens attached to the camera part of a smartphone. (**B**) A smartphone screen through an EHD lens when voltage is applied.

Finally, we employed the EHD lens on a smartphone as a demonstration for daily use. As shown in Figure 9, we fixed the EHD lens to the camera of a smartphone and tried to magnify the object. In this experiment, we used the same DC voltage source as presented in Figure S1. At 0 kV, the object was blurred, but when we applied the voltage, it became a little clear, and the object was magnified. The results show that the EHD lens can be integrated with some devices in our daily life, such as smartphones. Although the EHD lens requires high voltage, it is safe. In the case of electrical power, the parameter that affects people is not the voltage but the current. For example, the voltage of static electricity is about 4 kV, but this static electricity does not harm people. This is because the current output is very small. Humans feel electricity at currents on the order of mA, but our pumps are driven by currents on the order of μA [32].

## 5. Conclusions

In this study, we designed, fabricated, and developed a simple and thin focus-tunable liquid lens using an EHD pump. We composed this EHD lens from readily available components and can fabricate it at low cost. The proposed EHD lens consists of a polypropylene (PP) sheet for the exterior, a copper sheet for the electrode, and an acrylic elastomer for the fluidic channel where dielectric fluid and pure water are filled. The total dimensions of the EHD lens were 47 × 15 × 1.3 ${\mathrm{mm}}^{3}$. In this paper, we realized the EHD lens using a digital fabrication method, and constructed a numerical model to evaluate its performance. By applying a voltage, the radius of curvature of the lens changes and the area magnification of the object increases. At 0°, the EHD lens enlarged the object by about 185% at 2.5 kV applied. The EHD lens could be driven at the tilt angles of 90° and 180° due to the surface tension. The results show that the numerical model agrees well with the experimental results. Finally, we have shown that the EHD lens can be integrated with some devices in our daily life, such as smartphones. In future works, we will integrate our device using a simple high-voltage DC-DC converter. We expect that this study will contribute to the miniaturization of focus-tunable lenses.

## Supplementary Materials

The following are available online at https://www.mdpi.com/article/10.3390/mi12121452/s1, Figure S1: Experimental setup for the performance investigation of Ultra-thin Focus-tunable Liquid Lens.
