# Supplementary material for: A DIY Fabrication Approach for Ultra-Thin Focus-Tunable Liquid Lens Using Electrohydrodynamic Pump
Source: Micromachines (Basel). 2021 Nov 26;12(12):1452. doi: 10.3390/mi12121452 (PMC8706613; doi:10.3390/mi12121452)
Supplement: Supplementary file 1 [file micromachines-12-01452-s001.zip › micromachines-1464111-supplementary .pdf]

micromachines

Supplementary information

A DIY Fabrication Approach for Ultra-thin Focus-tunable Liquid Lens Using Electrohydrodynamic pump

Taichi Murakami [1,*], Yu Kuwajima [1], Ardi Wiranata [1,2], Ayato Minaminosono [1], Hiroki Shigemune [3], Zebing Mao[1,*], Shingo Maeda [1,*]

[1]Department of Mechanical Engineering, Shibaura Institute of Technology Tokyo 135-8548, Japan; md20077@shibaura-it.ac.jp(T.M.); nd21105@shibaura-it.ac.jp(Y.K.); nb19501@shibaura-it.ac.jp(A.W.); nb20108@shibaura-it.ac.jp(A.M.); zebingv5@shibaura-it.ac.jp(Z.M.); maeshin@shibaura-it.ac.jp(S.M.)
[2]Department of Mechanical and Industrial Engineering, Faculty of Engineering, University of Gadjah Mada, Jalan Grafika No. 2, Yogyakarta 55281, Indonesia.
[3]Department of Electrical Engineering, Shibaura Institute of Technology Tokyo 135-8548, Japan; hshige@shibaura-it.ac.jp(H.S.)
*Correspondence: md20077@shibaura-it.ac.jp(T.M.); zebingv5@shibaura-it.ac.jp(Z.M.)； maeshin@shibaura-it.ac.jp(S.M.)

To investigate the performance of Ultra-thin Focus-tunable Liquid Lens, we employ high precision DC voltage amplifier (HEOPT20B10 from Matsuda Inc.). Figure S1 depicts the complete experimental setup for the tunable liquid lens performance investigation. Currently, we focussed on establishing a simple fabrication approach for ultra-thin focus-tunable liquid lens using electrohydrodynamic pump. Then, in the future works we will integrate our device with a simple high voltage DC-DC converter for the ease of device operation.

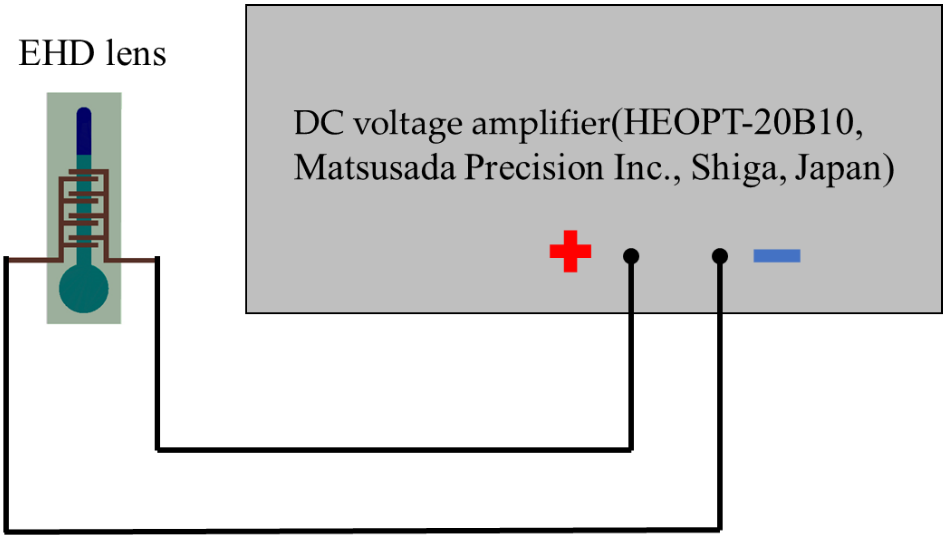

Figure S1. Experimental setup for the performance investigation of Ultra-thin Focus-tunable Liquid Lens.
